# Mental Health Refugees - Difficulties from the country of origin to the receiving country – A review

**DOI:** 10.1192/j.eurpsy.2022.1394

**Published:** 2022-09-01

**Authors:** D. Vila-Chã, B. Leal, I. Pinto, R. Mateiro, M. Avelino, J. Salgado

**Affiliations:** Centro Hospitalar Psiquiátrico de Lisboa, Psiquiatria, Lisboa, Portugal

**Keywords:** Refugees, global mental health

## Abstract

**Introduction:**

The most recent global refugee figures are staggering, with over 82.4 million people forcibly displaced as a result of persecution, conflict, violence, human rights violations. However, little is known about their long-term mental health.

**Objectives:**

This review aimed to assess prevalence of mental disorders and to identify the main factors associated with the development of mental disorders among refugees.

**Methods:**

We searched MEDLINE databases using the key terms “refugees” and “global mental health” without language or date restriction. Articles were considered for inclusion in the review if they comprised a population of refugees. Three studies were identified.

**Results:**

Our review showed a great heterogeneity in the prevalence of mental disorders that affect migrants showing an overall prevalence of 20% of these pathologies among them. War-related factors are more associated with Post Traumatic Stress Disorder and post-migration-related factors (acculturation, economic uncertainty and ethnic discrimination) are more associated with mood, anxiety and substance use disorders.

**Conclusions:**

Existing evidence suggests that mental disorders tend to be highly prevalent in refugees many years after resettlement. The increased risk is not only caused by the past adversities in the country of origin but also by the post-migration-related factors. Thus, there is a need for more consistent and rigorous research from a methodological point of view on the mental health of refugees, allowing to find measures to protect and promote their mental health.

**Disclosure:**

No significant relationships.

